# Learning faults in time: sequential behavioural modelling for complex fault detection in multi-robot systems

**DOI:** 10.3389/frobt.2026.1827739

**Published:** 2026-07-29

**Authors:** Faisal Firas Mazloum, David Portugal, Micael S. Couceiro

**Affiliations:** 1 Ingeniarius, Lda., Porto, Portugal; 2 Department of Electrical and Computer Engineering, Institute of Systems and Robotics (ISR), University of Coimbra, Coimbra, Portugal

**Keywords:** temporal behavioural modelling, multi-robot systems (MRS), exogenous fault detection, intermittent faults, actuator degradation, recurrent neural network (RNN), swarm robotics, distributed fault tolerance

## Abstract

Reliable fault detection in multi-robot systems requires models capable of capturing complex, time-dependent fault signatures that manifest over extended temporal horizons rather than instantaneous observations alone. Existing data-driven approaches operate reactively on behavioural snapshots, failing to capture fault modes whose discriminative signature depends on temporally ordered precursors. This work formalises a theoretical impossibility result demonstrating that memoryless classifiers are provably insufficient, and presents a Long Short Term Memory (LSTM) model operating on generic, primitive behavioural features to learn these latent temporal dependencies without domain-specific priors. Two complex fault types are evaluated: progressive actuator degradation and intermittent actuator dropout, each paired with a distinct swarm movement pattern representing varying levels of temporal complexity. Experimental results demonstrate that sequential models are essential when the fault signature is defined by ordered precursors, while memoryless models partially suffice for simpler temporal structures. Compute benchmarks confirm real-time feasibility for distributed onboard deployment.

## 1 Introduction

Reliable fault detection in multi-robot systems (MRS) operating in dynamic, unstructured settings requires models capable of capturing fault signatures across the full temporal spectrum, from sporadic and gradual degradation unfolding over extended operation to abrupt, instantaneous failures. Current exogenous fault detectors which exploit behavioural consensus across distributed robots to identify faulty neighbours without access to internal system states (Carminati et al., 2024; Khaldi et al., 2017; Tarapore et al., 2015; Tarapore et al., 2017; Tarapore et al., 2019) operate reactively on instantaneous behavioural snapshots, treating each observation as temporally independent. This architectural constraint renders them unreliable for two practically prevalent fault classes: (1) progressive actuator degradation, whose signature emerges gradually over extended operational horizons; and (2) intermittent faults, whose discriminative signature depends on a temporally ordered sequence of precursor events rather than the instantaneous system state. Non-data-driven approaches that model temporal dynamics (O’Keeffe, 2025) address some of these limitations but rely on explicit domain knowledge that are non-trivial to tune and have not been evaluated on complex fault patterns. This constraint limits opportunities for fault recovery and graceful degradation, effectively decreasing fleet uptime and increasing rescue and repair costs.

This work introduces a data-driven temporal behavioural model using recurrent neural networks (RNN), specifically Long Short-Term Memory modelling to overcome the above-mentioned limitations. This study formulates exogenous fault detection as a supervised learning problem, where each individual robot (agent) in the MRS predicts the fault state of its neighbouring robots based on a continuous history of behavioural features. The proposed approach is contrasted against a memoryless baseline (GBDT) operating on instantaneous feature vectors, selected to isolated the contribution of the recurrent architecture to fault discriminatory ability.

A natural alternative to the proposed memoryless baseline would be to provide a tabular learner (such as GBDT) with engineered temporal features: lagged values, sliding-window aggregates, or hand-designed motif descriptors. We deliberately do not pursue this configuration, primarily due to three reasons rooted in scalability, streaming flexibility, and the data-driven assumption itself.

*First, scalability.* Real-world fault manifestation horizons span hours to weeks for progressive degradation (Liu et al., 2012; Gao et al., 2021; Wang et al., 2018; Qiu et al., 2006); encoding such horizons as lag features inflates the input dimensionality to O(L⋅d) . Because this dimensionality scales directly with sequence length L , it introduces a computational deployment overhead that is incompatible with the memory budget of embedded robotic platforms (Lin et al., 2020). In contrast, gated architectures (such as LSTM) encode the same temporal information in a hidden state of constant size O(1) independent of sequence length (Hochreiter, 1997).

*Second, streaming flexibility.* Distributed exogenous detection involves variable-length observation sequences due to field-of-view dynamics, occlusions, and sensing dropout. These sequences also exhibit significant phase variability from temporal shifts or warping. For temporally complex faults such as intermittent faults, the discriminative motifs may appear at any temporal offset within the observable window. Although the ground-truth labels are phase-invariant, tabular learners utilising lag-feature representations (flattening windows in $R^{L\cdot d}$ ) are coupled to absolute positions, forcing the model to learn offset-specific representations, leading to generalisation degradation. Empirical evidence on the UCR archive directly demonstrates this failure mode: tabular classifiers operating on raw indexed representations of time-series suffer the largest accuracy degradation when phase misalignment is introduced, while phase-independent methods remain robust (Zhang et al., 2025b). In contrast, gated recurrent architectures have been shown to provide inductive bias: their learnable gates yield quasi-invariance to time transformations of the input, including dilations and phase misalignment that disrupts fixed-position dependencies (Tallec and Ollivier, 2018). Furthermore, the recurrence applies the same parametrised update at every step, $\left(h_{t},c_{t}\right)=g_{\theta }\left(h_{t-1},c_{t-1},f_{t}\right)$ with θ shared across all t , so the model’s response to a precursor motif depends on the motif’s content and historical context rather than fixed-window indices.

*Third, the data-driven assumption*. This assumption constrains the space of admissible baselines for this study. Benchmark tests show that tabular learners can match or exceed neural network based performance when provided sufficiently informative features (Grinsztajn et al., 2022). However, designing such domain-specific features that encode the discriminative motif requires prior knowledge of the fault’s causal signature. Data-driven representation learning is intended to discover this exact pattern directly from data (Bengio et al., 2013), a task where neural networks inherently excel (Lipton et al., 2015; Lim and Zohren, 2021; Verner, 2019). If the signature were *a priori* known, a rule-based or threshold-based detector would suffice and no learning would be required. Generic, domain-agnostic aggregates or statistical features (e.g., tsfresh (Christ et al., 2018), catch22 (Lubba et al., 2019), rolling mean) avoid this domain-specific design, but are predominantly invariant to ordering. Intermittent fault case studies across discrete event systems, networked architectures, and concurrent software have been formally characterised as dependent on ordered precursor sequences (Soldani et al., 2006; Contant et al., 2002; Sotiropolos and Vassilakis, 2020; Sotiropoulos and Vassilakis, 2025), motivating the use of data-driven models capable of representing temporal ordering rather than aggregated, statistical, or order-invariant feature representations.

While a comparative study of lagged, aggregate, and other engineered temporal features against sequential models on primitive inputs is an interesting direction for future works, this study targets a prior gap: existing exogenous fault detection studies in multi-robot systems are evaluated only on simple or abruptly-manifesting faults (Carminati et al., 2024; Khaldi et al., 2017; Tarapore et al., 2015; Tarapore et al., 2017; Tarapore et al., 2019), leaving complex time-dependent fault types unaddressed. Accordingly, the following research questions evaluate two model architectures that differ only in their access to temporal information:

1. Research Question 1 (RQ1): Are memoryless classifiers sufficient for reliable exogenous fault detection of persistent time-dependent faults such as progressive actuator degradation in distributed robot systems?
2. Research Question 2 (RQ2): Are memoryless classifiers sufficient for reliable exogenous fault detection of sporadic, time-dependent faults such as intermittent fault in distributed robot systems?
3. Research Question 3 (RQ3): Does a sequential model (LSTM) with temporal modelling overcome the limitations of memoryless classifiers for reliable exogenous fault detection across both fault types?

This paper is organised as follows: Section 2 reviews the related work; Sections 3, 4 describes the proposed method, fault models, and experimental design and training procedures; Section 5 presents the comparative results of each model and discusses the study’s implications; Section 6 concludes the paper, outlining limitations and directions for future research.

## 2 Review of related literature

The rising popularity of distributed MRS stems from several advantages that have been demonstrated in empirical studies, highlighting the distributed nature of the robotic system that eliminates the single-point-of failure, a determinate consequence of centralised architectures. This creates inherent robustness, scalability, and flexibility across a wide range of missions and robot fault categories (Miller and Gandhi, 2021; Winfield and Nembrini, 2006; Zhang et al., 2025a). Despite the above-mentioned advantages, several studies demonstrate that partial failures that manifest in individual robots in the system (e.g., actuator faults) can dramatically degrade collective motion and deteriorate global mission performance. This highlights the need for distributed, exogenous methods for more reliable fault tolerance to maintain autonomy and safeguard mission success (Winfield and Nembrini, 2006; Bjerknes and Winfield, 2013).

In multi-robot systems, fault tolerance is generally outlined as a three-stage pipeline: (1) Fault detection (FD) which is concerned with identifying when a fault occurs; (2) Fault isolation (FI) for locating the component that deviates from identical neighbouring components or hardware models; and (3) Fault recovery (FR), an action phase to mitigate fault propagation and restore stability in the system. The literature can be grouped by where the analysis is performed (endogenous vs. exogenous) (O’Keeffe et al., 2017) or by the framework used such as, but not limited to: Fault detection and diagnosis (FDD) or fault detection, diagnosis, and recovery (FDDR) (O’Keeffe, 2025). The frontier in exogenous fault tolerant MRS involve detection and diagnosis (FDD), most notably using: (1) immune-inspired or (2) ML-based FDD frameworks.

Bio-inspired methods for fault tolerant MRS draw inspiration from natural immune systems to enable distributed and adaptive fault detection in neighbouring robots. An example of such is the cross-regulation model (CRM) introduced by Tarapore et al. (Tarapore et al., 2015; Tarapore et al., 2017), which mimics how the immune system distinguishes self cells from abnormal/foreign cells through the interaction of T-cells on the same antigen-presenting cells (APCs). A fault is detected in a behavioural feature vector (BFV) when the total population of effector cells exceeds that of regulatory cells $\left(T_{E}>T_{R}\right)$ , otherwise normal/tolerant $\left(T_{R}>T_{E}\right)$ . A key benefit in this method is adaptive learning: the CRM’s biological foundation induces adaptivity without hard-coded priors of normality, enabling task-agnostic fault tolerant swarms, i.e., robustness across task transitions as demonstrated through numerous swarm primitive behaviours (Tarapore et al., 2015). In spite of this inherent adaptivity, the model’s efficacy remains tied to explicit domain knowledge. This creates a bottleneck, as the system still requires careful architectural parameter selection and non-primitive feature design to characterize expected behaviours, necessitating prior information about the environment each agent will be subjected to, the actions it will perform, and how it responds to events. Beyond these design constraints, there is a further limitation regarding the model’s temporal response. Because the underlying BFVs are engineered to detect abrupt faults, the system operates reactively rather than proactively.

To address some of these limitations, specifically the reliance on meticulous feature engineering and the reactive nature of detection, O’Keeffe (O’Keeffe, 2025) introduced the Artificial Antibody Population Dynamics model (AAPD). Derived from Farmer et al.’s mathematical model of antibody population dynamics in the natural immune system (Farmer et al., 1986), AAPD shifts the focus towards anticipating faults before catastrophic field failure. The system functions by encoding robot behaviour as temporal paratopes $p_{i}$ over fixed-length windows of primitive behavioural features, which are added to local repertoires (X) of known distinct paratopes, analogous to immune memory. This mechanism mimics the natural immune system’s ability to distinguish self from non-self based on antigen abundance and persistence based on population dynamics. By detecting gradual faults before complete failure, AAPD enables predictive maintenance scheduling, allowing the faulty robots to trigger fault recovery autonomously. However, several architectural limitations constrain the model’s applicability. First, it still relies heavily on domain expertise for parameter tuning, with values failing to transfer well across other fault types within the same system. Similar to CRM, AAPD (in its unsupervised configuration) presupposes majority behavioural cohesion to establish a normal baseline, making it prone to high-false positive rates in tasks with high behavioural stochasticity. Finally, the model’s fault detection operates via cross-correlation residual matching between stored paratopes and current window L that does not generalize beyond previously encountered fault sequences, i.e., complex faults whose expression relies on temporal ordering (e.g., intermittent faults) may have substantial variance on raw feature profiles across sequences, requiring AAPD to have stored a matching exemplar for each distinct variant.

Contrarily, data-driven techniques shift the burden of fault detection from domain-specific engineering to learned representations or derived statistics (Carminati et al., 2024; O’Keeffe et al., 2018; Khaldi et al., 2017; Lau et al., 2011). Statistical approaches offer lightweight, interpretable detection without requiring labelled fault data (Khaldi et al., 2017; Lau et al., 2011). Machine learning approaches go further, ingesting high-dimensional telemetry, such as robot velocities and inter-robot distances to implicitly learn the latent signatures of healthy versus faulty robots, generalizing across fault types and varying ratios of faulty robots (Carminati et al., 2024), a critical limitation in bio-inspired methods where a normal baseline is contingent on majority behaviour rather than a known fault signature. However, these approaches are trained to flag instantaneous or abrupt faults, and have not been validated on faults that manifest over time. This gap limits fault recovery options, hindering global mission performance (O’Keeffe, 2025), as well as increasing the unreliability of the model under complex, sporadic fault types whose fault signature is dependent on temporally ordered sequence of events. This motivates the adoption of data-driven temporal modelling solutions, capable of encoding time-series to accumulate historical evidence to identify time-dependent, complex fault types.

Within the broader family of temporal classifiers, the alternatives, notably classical recurrent neural networks (RNNs), gated recurrent units (GRUs), temporal convolution networks (TCNs), transformers, and hidden Markov models (HMMs), each presents trade-offs and limitations relevant to distributed fault detection. Among the gated architectures, LSTM and GRU show no consistent edge across benchmarks: GRUs are more parameter-efficient, while LSTMs provide more stable performance for increasing temporal complexities in time-series data (Cahuantzi et al., 2023; Yunita et al., 2025). Both are treated as strong recurrent baselines in comparative deep time-series studies, with plain RNNs underperforming in longer horizons due to vanishing gradient instability (Yunita et al., 2025; Dos Santos et al., 2025). Beyond recurrent models, Transformers have been demonstrated to be powerful alternatives; however, impose critical deployment limitations for real-time, resource-constrained platforms: the self-attention layers impose quadratic time and space complexity $\left(O\left(L^{2}\right)\right)$ with sequence length and consume substantially higher floating point operations (FLOPs) in a single forward pass compared to recurrent models (Hasan et al., 2025; Sarkar and Chu, 2025). As per HMMs, although they have been shown to thrive in data-scarce settings due their smaller parameter space, gated architectures such as LSTMs, achieve superior performance on more temporally intricate tasks given sufficient labelled data, with accuracy increasing as model complexity increases (Tadayon and Pottie, 2020; Liu et al., 2019). Furthermore, HMMs rely on first-order Markov assumption, which presupposes that future states depend solely on the current state, therefore neglecting long-range temporal dynamics where historical context is critical Petropoulos et al. (2017). While extensions to higher-order HMMs can recover some long-range dependencies, they do so at exponentially increasing computational cost (Liao et al., 2021; Petropoulos et al., 2017). Head-to-head comparisons of TCNs and LSTMs show no consistent winner across domains, and that both architectures can successfully tackle the same time-series tasks with marginal and task-dependent differences in accuracy and convergence speed (Wardani et al., 2025; Li et al., 2025; Gopali et al., 2021). Although alternative and optimised temporal architectures may offer competitive or superior performance on specific tasks, no consistent winner emerges across the broader benchmarking literature. We therefore adopt LSTM as the representative temporal architecture, an established and competitive baseline for evaluating the central hypothesis of this work.

This study revolves around exogenous fault detection: detecting faults in neighbouring robots using only externally observable behaviour and exchange messages, without access to internal sensor readings (Carminati et al., 2024; Tarapore et al., 2015; 2017; Miller and Gandhi, 2021). This requires the temporal architecture and features to be expressive enough to model the codomain using limited, higher-level, generic inputs. To assess the feasibility of constructing a temporal model using generic features, recurrent neural networks (RNNs), namely, long short term memory (LSTM) models have demonstrated the ability to process behavioural features such as joint positions and velocities to estimate joint torque at the current timestep (Huang et al., 2024). In the context of swarm robotics, LSTM models have been used to capture collective intelligence by processing windowed sequences of agents states. By feeding the sequential network a historical buffer of primitive features such as agent positions and velocities, these models output a set of future state vectors, demonstrating multi-step predictions and imitations of swarm dynamics (Zhou et al., 2019). These studies demonstrate that LSTMs have the capacity to process a limited and primitive input set to output accurate predictions, and that generic, primitive observable features are expressive enough to characterize swarm agent behaviours. This exogenous and generic approach ensures that the detection logic is not tied to a specific hardware configuration, but rather to the fundamental patterns of motion and interaction characterizing the movement behaviour of the swarm.

## 3 Methodology

This study proposes a data-driven temporal model for exogenous fault detection in a distributed MRS experiencing complex, time-dependent fault types. The methodology is organised as follows:

- What is observed (Section 3.1). Each robot $r_{i}$ observes the behaviour of its neighbour $r_{j}\in N_{i}\left(t\right)$ using exteroceptive RAB sensors, computing a set of generic, primitive behavioural features which serve as inputs to both model architectures. The output is a binary fault label (healthy vs. faulty) per robot per timestep.
- Why sequential modelling is necessary (Section 3.2). We formalize that memoryless classifiers are insufficient for fault types whose discriminative signature depends on temporally ordered precursors, and that LSTM is expressive enough to characterise the behavioural fault signature using primitive, observable features via exteroceptive sensors.
- What fault modes are modelled (Section 3.3). Two fault modes are evaluated, each representing a distinct class of real-world actuator fault modes with non-trivial temporal structure: progressive actuator degradation, a persistent and monotonically worsening fault; and intermittent actuator dropout, a sporadic, self-recovering fault whose onset is governed by latent, ordered sequence of events.
- What swarm movement patterns are used (Section 3.4). Each fault type is paired with a swarm movement pattern chosen to represent varying levels of temporal complexity: progressive actuator degradation is evaluated under swarm aggregation, a deterministic primitive behaviour; and intermittent fault evaluated under adaptive sampling, a stochastic multi-modal pattern introducing high temporal ambiguity. These pairing ensure that the temporal challenge faced by the models reflect the fault type’s inherent complexity rather than behavioural noise.

Furthermore, the temporal models evaluated are data-driven and use generic behavioural measurements as inputs, requiring no domain-specific priors, making the approach applicable beyond the specific platform and mission evaluated here.

### 3.1 Behavioural features and fault labels

#### 3.1.1 Input space

This work adopted the numerical feature set proposed by Carminati et al. (2024), which describes the observable behaviour of each neighbouring robot through five primitive measurements derived from local RAB sensors, requiring no domain-specific engineering or access to internal state of its neighbours.

At each timestep t , the observing robot $r_{i}$ computes the behavioural feature vector $x_{j}\left(t\right)\in R^{5}$ for each of its neighbouring robots, known as observed robots $r_{j}\in N_{i}\left(t\right)$ , defined as:

$$x_{j}\left(t\right)={\left[f_{j}^{1}\left(t\right),\;f_{j}^{2}\left(t\right),\;f_{j}^{3}\left(t\right),\;f_{j}^{4}\left(t\right),\;f_{j}^{5}\left(t\right)\right]}^{⊤}\in R^{5}\text{,}$$ (1)

where $f_{j}^{1}$ and $f_{j}^{2}$ describe the left and right thruster speeds of robot $r_{j}$ , communicated directly to neighbouring robots. $f_{j}^{3}$ describes the distance from $r_{j}$ to its nearest neighbour, and $f_{j}^{4}$ denotes the average distance from $r_{j}$ to all its neighbours, both estimated by robot $r_{i}$ using the Law of Cosines applied to its local RAB measurements. Lastly, $f_{j}^{5}$ describes the accumulated distance travelled by $r_{j}$ over the last $W_{l}$ seconds, estimated using the observed thruster speed history.

The features are sampled at Δt s, extracting one observation per second per observed neighbour. The input space varies by model architecture. The non-sequential model (GBDT) treats every observation at time t as independent, therefore creating an instantaneous behavioural feature vector as described in Equation 1, whereas the sequential model (LSTM) compresses continuous observations into a fixed temporal window of length L (Equation 2), varying by task (described in Section 4.2):

$$X_{j}\left(t\right)={\left[x_{j}\left(t-L+1\right),\;x_{j}\left(t-L+2\right),\ldots ,\;x_{j}\left(t\right)\right]}^{⊤}\in R^{L\times 5}\text{.}$$ (2)

The feature representation of sequential models enables temporal patterns to be learned implicitly purely based on data, requiring minimal domain knowledge: no hand-crafted thresholds, features, or behaviour specific-parameters are embedded into the feature space.

#### 3.1.2 Output space

Both model architectures are tasked with binary fault classification, producing a fault label $y_{j}\left(t\right)\in \left\{0,1\right\}$ for each observed robot $r_{j}$ at time t , where $y_{j}\left(t\right)=1$ indicates a robot experiencing a fault: degradation or intermittent fault, and $y_{j}\left(t\right)=0$ indicates a healthy robot exhibiting expected behaviour. Each model initially produces a fault probability ${\hat{p}}_{j}\left(t\right)\in \left[0,1\right]$ which is then thresholded at the optimal value $p_{\text{thresh}}^{*}$ (described in Section 4.3) to obtain a binary label (Equation 3):

$${\hat{y}}_{j}\left(t\right)=1\left({\hat{p}}_{j}\left(t\right)\geq p_{\text{thresh}}^{*}\right)\text{.}$$ (3)

For the sequential model (LSTM), a many-to-one labelling scheme is used to apply a window-level label on the entire sequence $X_{j}\left(t\right)$ , corresponding to the ground truth label at the final timestep: $Y_{j}\left(t\right)=y_{j}\left(t\right)$ . This ensures that the fault is captured at the earliest detectable instance.

Although the fault labelling strategy is consistent across both tasks, i.e., each extracted sample inherits the label of the latest timestep observation, the fault status varies by task and fault type, described in detail in Section 3.3, 4.2.

### 3.2 Justification for sequential modelling

In this work, both the Gradient Boosting Decision Tree (GBDT) baseline and the proposed LSTM-based model operate on the same five behavioural features introduced by Carminati et al. (2024), which are generic, primitive observable features. Let $f_{t}=\left(f_{1,t},f_{2,t},f_{3,t},f_{4,t},f_{5,t}\right)\in R^{5}$ denote the feature vector at time t , where the components encode, for example, left and right thruster speeds, nearest and average neighbour distances, and distance travelled over a fixed window.

Despite using identical features, the two models differ in how they access temporal information. The GBDT implements a *memoryless* classifier (Equation 4)

$$h_{\text{GBDT}}:\;R^{5}\to \left\{0,1\right\},\;{\hat{y}}_{t}=h_{\text{GBDT}}\left(f_{t}\right),$$ (4)

which maps instantaneous observations to a fault label. Contrarily, the LSTM implements a *sequence* classifier (Equation 5)

$$h_{\text{LSTM}}:\;R^{5\times L}\to \left\{0,1\right\},\;{\hat{y}}_{t}=h_{\text{LSTM}}\left(f_{t-L+1},\ldots ,f_{t}\right),$$ (5)

which can use the evolution of the same features over a finite history of length L .

#### 3.2.1 Problem construction and label definition

We define the ground-truth fault label at time t to depend on both the current thruster speeds and a specific temporal precursor pattern in the recent behavioural history. Let.

-
$Z_{t}$ be the event the robot has zero thruster speed at time t , expressed in terms of the components of $f_{t}$ ;
-
$P_{t}$ be the event that, within the last L seconds, the robot executed the behavioural sequence *turn left* → *turn right* → *turn left* → *enter a new zone*, as encoded in the sequence ${\left(f_{\tau }\right)}_{\tau \in \left[t-L,\;t\right)}$ .

The true label $y_{t}\in \left\{0,1\right\}$ (Equation 6) is then defined as

$$y_{t}=\left\{\begin{array}{cc} 1, & \text{if }Z_{t}\text{ and }P_{t}\text{ both hold},\\ 0, & \text{otherwise}. \end{array}\right.$$ (6)

Both healthy and faulty robots may exhibit zero thruster speeds, however, a zero-speed event is classified as faulty if and only if it is preceded by the specified precursor sequence in the recent behavioural history.

#### 3.2.2 Impossibility result for memoryless classifiers

We first show that no memoryless classifier that depends only on the instantaneous feature vector $f_{t}$ can represent the labelling rule (6) on all admissible trajectories.

Let $H_{0}=\left\{h:R^{5}\to \left\{0,1\right\}\right\}$ denote the hypothesis class of all memoryless classifiers acting on $f_{t}$ . By construction of our simulator, there exist two trajectories of feature vectors, $\left(f_{1},\ldots ,f_{t}\right)$ and $\left(f_{1}^{\prime },\ldots ,f_{t}^{\prime }\right)$ , such that:

1. The instantaneous features at time t coincide, $f_{t}=f_{t}^{\prime }$ , so that both robots have identical thruster speeds and local swarm interaction statistics at that time.
2. Only the second trajectory satisfies the precursor condition in the last 50 s, i.e., $P_{t}$ holds for $\left(f_{\tau }^{\prime }\right)$ but not for $\left(f_{\tau }\right)$ .

From the label definition in (6), it follows that

$$y_{t}=0,\;y_{t}^{\prime }=1,$$

where $y_{t}=f^{*}\left(f_{1},\ldots ,f_{t}\right)$ and $y_{t}^{\prime }=f^{*}\left(f_{1}^{\prime },\ldots ,f_{t}^{\prime }\right)$ denote the true labels for the two trajectories.

Proposition 1 (Insufficiency of Memoryless Classifiers). *Under the labelling rule* (6)*, there is no function* $h\in H_{0}$ *such that* $h\left(f_{t}\right)=y_{t}$ *for all admissible trajectories* $\left(f_{1},\ldots ,f_{t}\right)$ .

Proof. Consider the two trajectories described above, with $f_{t}=f_{t}^{\prime }$ but $y_{t}\neq y_{t}^{\prime }$ . Any $h\in H_{0}$ satisfies

$$h\left(f_{t}\right)=h\left(f_{t}^{\prime }\right),$$

because its input at time t is identical in both cases. Hence it is impossible for h to satisfy simultaneously $h\left(f_{t}\right)=y_{t}$ and $h\left(f_{t}^{\prime }\right)=y_{t}^{\prime }$ when $y_{t}\neq y_{t}^{\prime }$ . Therefore no such h can coincide with the true labelling function $f^{*}$ on all admissible trajectories.

Proposition 1 implies that, even though the GBDT operates on the same features $f_{1},\ldots ,f_{5}$ as the LSTM, its hypothesis class is representationally insufficient for our precursor-based fault detection task, because it has no access to the temporal context in which zero-speed events occur.

#### 3.2.3 Expressivity of the LSTM hypothesis class

We now argue that the LSTM hypothesis class is, in principle, expressive enough to implement the labelling rule (6). The precursor condition $P_{t}$ can be captured by a finite-state automaton whose states encode which prefix of the sequence “left → right → left → zone entry” has been observed within the last 50 s. At each time step, this automaton reads the current feature vector $f_{\tau }$ , updates its state according to the observed primitive behaviour, and resets when the L s horizon is exceeded.

Recurrent neural networks are known to be capable of simulating finite-state automata by encoding the automaton state in their hidden units and updating it based on the input sequence. This has been formalised for various RNN architectures, and LSTMs are at least as expressive in this sense. Consequently, there exists an LSTM function (Equation 7)

$$h_{\text{LSTM}}^{*}\in H_{\text{LSTM}}$$ (7)

such that, for any admissible trajectory (Equation 8),

$$h_{\text{LSTM}}^{*}\left(f_{t-L+1},\ldots ,f_{t}\right)\;=\;f^{*}\left(f_{1},\ldots ,f_{t}\right)\;=\;y_{t}.$$ (8)

Combining Proposition 1 with this expressivity argument, we obtain a theoretical motivation for using an LSTM-based sequential model: with the same behavioural features $f_{1},\ldots ,f_{5}$ , a memoryless model such as GBDT cannot, by construction, represent the true labelling function for our task, whereas an LSTM operating on the temporal evolution of these features can.

### 3.3 Fault modelling

Two fault types are evaluated in this study, each representing a distinct class of real-world actuator failure with non-trivial temporal structures in the observed behavioural signals.

### 3.3.1 Task I: progressive actuator degradation

This fault type addresses the gradual decline in thrust capacity or motor efficiency, as caused by ESC damage or bearing wear (Gorospe et al., 2017; Hamani et al., 2025), or in the REMORA use-case, biofouling accumulation on the USV’s thrusters (Valchev et al., 2022; Song et al., 2020). Marine growth such as barnacles and algae coat USV thrusters, increasing surface roughness on propeller blades which increases hydrodynamic drag, effectively reducing its thrust. Biofouling develops gradually over days or weeks. In this study, the degradation rate is accelerated to enable controlled experimental evaluation.

The actuator degradation is modelled as a linear, symmetric decline in motor capacity applied equally to both thrusters, where the commanded speed is scaled by the degradation coefficient defined as (Equation 9):

$$\gamma \left(t\right)=\max\left(0,1-\delta \cdot \left(t-t_{\text{inject}}\right)\right)\in \left[0,1\right],\;t\geq t_{\text{inject}},$$ (9)

where $t_{\mathrm{inject}}$ denotes the fault injection time, and δ denotes the degradation rate. Complete failure occurs at $t_{\mathrm{inject}}+\tau$ , at which point γ=0 , and the robot becomes fully immobilised. At each timestep, the commanded left and right thruster speeds are scaled by γ(t) , therefore degrading both actuators symmetrically. Since degradation is monotonically progressive, the fault state persists and is assigned to all timesteps of the observed robot $r_{j}$ , following any measurable degradation, specifically $y_{j}\left(t\right)=1\left(t\geq t_{\mathrm{inject}}\right)$ .

Given that faulty robot’s actuators degrade monotonically, the remaining useful life (RUL), representing the amount of time until complete actuator failure v=0 can be represented as (Equation 10):

$$\text{RUL}\left(t\right)=100\cdot \max\;\left(0,\;1-\delta \cdot \left(t-t_{\text{inject}}\right)\right),\;t\geq t_{\text{inject}}\text{,}$$ (10)

where an RUL=100 indicates a robot that has not yet experienced any visible degradation, therefore is healthy.

#### 3.3.2 Task II: intermittent dropout

This fault type addresses intermittent faults, a fault class characterised by sporadic, self-recovering failures that recur due to the same underlying pattern (Contant et al., 2002). Unlike permanent faults which are persistent, intermittent faults exhibit a dynamic state that alternates between faulty and recovered, making it fundamentally harder to detect (Contant et al., 2002; Soldani et al., 2006). A constituent property of intermittent faults across domains is that their expression depends on a temporal precursor, i.e., the current instantaneous system state is dependent on the sequence of events that preceded it. This property has been formally defined in discrete event systems (Contant et al., 2002), networked architectures (Soldani et al., 2006), and concurrent software Sotiropolos and Vassilakis (2020); Sotiropoulos and Vassilakis (2025). This property is precisely what renders memoryless classifiers provably insufficient (Proposition 1). This is consistent with findings that intermittent faults manifest as multivariate subsequence anomalies where temporal continuity is critical for reliable detection and evaluation, and deep architectures sensitive to sequential structures are more affected by temporally consistent validation schemes than shallow learners that rely on aggregated features (Hespeler et al., 2025). These findings may suggest that latent temporal patterns critical for reliable fault detection and prognosis are preserved in raw sequential observations and that the ordered sequential structure of precursor events is a defining and discriminative characteristic of intermittent faults that distinguishes them from transient, permanent, or progressive fault modes.

We model an electronics speed controller (ESC) safety-induced system shutdown, a temporary, self-recovering loss of motor thrust caused by protective shutdown. While ESC-specific intermittent fault case studies are scarce, intermittent electrical continuity faults such as loose or chafed conductors that sporadically open, short, or arc, are a well documented cause of temporary power and communication disruption in electrical control systems that self-recovers upon mechanical or electrical stabilisation (Smith et al., 2008; Ahmad et al., 2014; Correcher et al., 2010; Kim and Johnson, 2006). Mechanical vibrations and oscillations, such as those produced by wave motion, motor operation, or directional manoeuvers, are established causes of intermittent signal dropout, where fretting-induced periodic contact gap formation lead to temporary electrical discontinuity (Maul et al., 2002; Fu et al., 2012). ESC degradation under sustained high-current operation has also been shown to incur performance deteriorations and a potential loss of thrust (Gorospe et al., 2017), and a protective ESC shutdown is a standard safety response to such situations. Together, these mechanisms establish that intermittent actuator dropout (manifesting as ESC shutdown) is physically plausible under the combined effects of high-load operation and mechanical oscillation in marine robotic platforms.

The fault is therefore modelled as depending on an ordered behavioural precursor, reflecting the established findings that these faults are characterised by a temporal sequence of events rather than instantaneous state observations alone (Contant et al., 2002; Soldani et al., 2006; Sotiropolos and Vassilakis, 2020; Sotiropoulos and Vassilakis, 2025). While the specific precursor pattern is a synthetic modelling choice, it is consistent with the above-mentioned physically motivated mechanisms, and the latent, unknown nature of real fault signatures motivates data-driven models that implicitly learn these temporal dependencies.

In the adaptive sampling mission, robots in high-complexity zones sustain operation with higher load to perform focused or high-resolution sampling of the water conditions. This induces high-torque demand, elevating motor current draw, contributing to thermal accumulation, while repeated directional manoeuvres and an instant ramp up in motor velocities introduce mechanical oscillations that can destabilise electrical connectivity. These events lead to temporary, protective shutdown, manifesting as a temporary loss in thrust. Upon stabilisation, the system self-recovers to nominal operation, highlighting the intermittent cycle of fault onset → self-recovery → recurrence. Formally, a faulty robot $r_{j}$ enters an episode of ESC shutdown if and only if the following sequence of events occurs in strict temporal order:

1.
$r_{j}$ has continuously operated in high-resolution sampling mode for a cumulative duration of $T_{\text{high}-\text{res}}$ s, leading to sustained high-torque operation and consequently to a higher current draw, consistent with documented ESC thermal stress under sustained high-torque operation (Gorospe et al., 2017).
2.
$r_{j}$ performs any of the following turn sequences while in high-resolution sampling mode: [L,R,L] or [R,L,R] , where L and R represent left and right turns, observable through thruster speeds $f_{j}^{1}\left(t\right)$ and $f_{j}^{2}\left(t\right)$ , respectively. This criterion represents the synthetic precursor pattern, where repeated directional manoeuvrers introduce oscillations consistent with documented case studies (Contant et al., 2002; Sotiropolos and Vassilakis, 2020; Soldani et al., 2006). While deterministic in nature, the complexity of the pattern produces high epistemic uncertainty, appearing stochastic in the absence of temporal context.
3.
$r_{j}$ transitions to a lower complexity sampling zone, entering the investigation or survey mode which requires a higher USV motor speed operation. This rapid increase in motor velocity further contributes to mechanical oscillations that impose additional electrical instability (Contant et al., 2002; Sotiropolos and Vassilakis, 2020; Soldani et al., 2006).

Upon satisfaction of all three conditions, an ESC shutdown episode is triggered at time $t_{0}$ . The motor output speeds $v_{l,f}\left(t\right)$ are attenuated for a duration of $\Delta t_{\text{dropout}}$ as follows (Equation 11):

$$v_{l,f}\left(t\right)=\left\{\begin{array}{cc} 0\text{,} & t_{0}\leq t<t_{0}+\Delta t_{\text{dropout}}\\ v_{\text{nom}}\text{,} & \text{otherwise.} \end{array}\right.$$ (11)

The ground truth label $y_{j}\left(t\right)$ is assigned based on this interval to indicate the system state (Equation 12):

$$y_{j}\left(t\right)=\left\{\begin{array}{cc} 1,\; & t_{0}\leq t<t_{0}+\Delta t_{\text{dropout}}\;\left(shutdown\right)\\ 0,\; & \text{otherwise}\;\left(Healthy\right). \end{array}\right.$$ (12)

The complete intermittent fault condition, consolidating all three precursor requirements and the active dropout event at time t is formally expressed as (Equation 13):

$$\begin{array}{l} F_{\text{IF}}\left(r_{j},t\right)=1\left[\underset{\left(i\right)cumulativehigh-resolutionoperation}{\underbrace{\int_{t-T_{\text{thresh}}}^{t}1\left[M\left(r_{j},s\right)=\text{HR}\right]\;ds\geq \Delta t_{\text{high}-\text{res}}}}\wedge \underset{\left(ii\right)precursorturnsequence}{\underbrace{\psi_{j}^{\text{turn}}\in \left\{\left[L,R,L\right],\left[R,L,R\right]\right\}}}\right.\wedge \\ \;\left.\underset{\left(iii\right)modetransition}{\underbrace{M\left(r_{j},t^{-}\right)\neq M\left(r_{j},t^{+}\right)}}\wedge \underset{\left(iv\right)activedropoutatt}{\underbrace{v_{l,r}^{j}\left(t\right)\approx 0}}\right]\text{.} \end{array}$$ (13)

where $F_{\text{IF}}\left(r_{j},t\right)\in \left\{0,1\right\}$ is the intermittent fault indicator for $r_{j}$ at time t , $M\left(r_{j},s\right)$ denotes the sampling mode of $r_{j}$ at time s , HR represents high-resolution sampling mode, $M\left(r_{j},t^{-}\right)$ and $M\left(r_{j},t^{+}\right)$ represent the instants immediately before and after a mode transition, $T_{\text{high}-\text{res}}$ represents the cumulative high-resolution operation duration threshold, $\psi_{j}^{\text{turn}}$ represents the last three recent turn sequence executed by $r_{j}$ , and $v_{l,r}^{j}\left(t\right)$ for the left and right thruster speeds of $r_{j}$ at time t .

### 3.4 Movement patterns

In this work, two swarm movement patterns are evaluated, each representing a distinct behavioural pattern and level of temporal complexity in the observed behavioural feature space.

#### 3.4.1 Aggregation pattern

**FIGURE 1:**
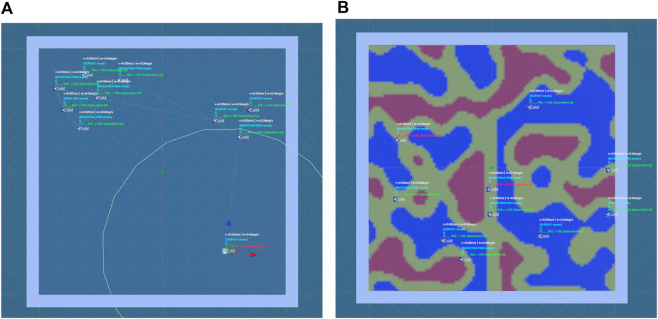
Example of swarm movement patterns. **(A)** Aggregation pattern showing convergent robot formation. **(B)** Adaptive sampling pattern showing varied spatial complexity. Healthy robots are highlighted with green gizmos, and red for faulty robots.

The aggregation swarm movement pattern is a primitive movement behaviour wherein robots move towards the centroid of observable neighbours (see Figure 1A), transitioning between four deterministic states based on spatial configurations and temporal constraints. Let $S_{j}\left(t\right)\in \left\{1,2,3,4\right\}$ denote the current state of the observed robot $r_{j}$ at time t , and $f_{j}^{3}\left(t\right)$ represent the nearest neighbour from $r_{j}$ as observed by $r_{i}$ . State transitions are dependent on the cohesion and separation thresholds $d_{\mathrm{coh}}$ and $d_{\mathrm{sep}}$ respectively, as well as a cohesion duration timer $\Delta \tau_{j}\geq 0$ . The expected left and right thruster speeds $E\left[v_{j}\left(t\right)\right]$ are fully determined by the active state $S_{j}\left(t\right)$ (Equation 14):

$$E\left[v_{j}\left(t\right)\right]=\left\{\begin{array}{cc} v_{\max}\text{,} & \text{if }S_{j}\left(t\right)\in \left\{1,2,3\right\}\;\left(explore/aggregate/disperse\right)\\ 0\text{,} & \text{if }S_{j}\left(t\right)=4\;\;\;\left(cohesion\right), \end{array}\right.$$ (14)

where state transitions are triggered by spatio-temporal conditions (Equation 15):

$$S_{j}\left(t+1\right)\neq S_{j}\left(t\right)\;⟺\;\left(f_{j}^{3}\left(t\right)>d_{\text{sep}}\right)\vee \left(f_{j}^{3}\left(t\right)\leq d_{\text{coh}}\right)\vee \left(\Delta \tau_{j}=0\right)\text{.}$$ (15)

While state transition is deterministic, a single instantaneous observation may not be fully sufficient to differentiate between states with identical behavioural features, such as stationary cohesion versus a complete actuator failure within cohesion distance. Instead, the active state and fault status can be determined by statistical aggregates, such as the accumulated distance travelled or mean velocities, calculated over a window. Because these descriptive statistics sufficiently capture the state dynamics, the specific temporal ordering of observations within the window is not critical for fault detection, making this pattern amendable to both sequential and non-sequential models, and serving as the simpler temporal problem of the two experimental tasks evaluated in this study.

**FIGURE 2:**
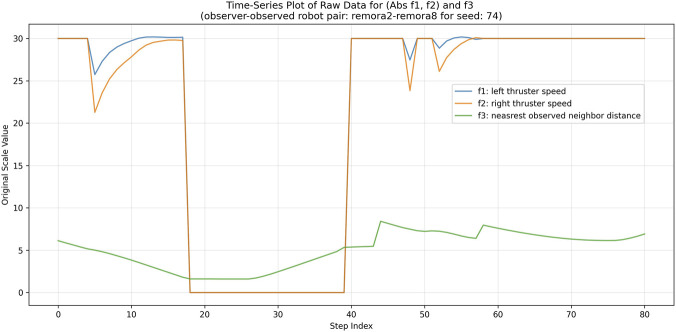
Time-Series plot of features $\left(f_{j}^{1},f_{j}^{2},f_{j}^{3}\right)$ under aggregation pattern. Thruster speeds are determined by the robot state. At t≈19 s, $r_{j}$ is within the cohesion distance $\left(f_{j}^{3}\leq d_{\text{coh}}\right)$ , triggering STAY_IN_GROUP state where robots remain stationary for a predefined duration. All other states involve $r_{j}$ moving at max velocity $\left(\left|v_{\max}\right|\right)$ .

The simplicity of the pattern is demonstrated by plotting the time-series distribution of the its features as shown in Figure 2, where thruster velocities take on discrete values: $v_{l,r}=0$ if $S_{j}\left(t\right)=4$ , and $|v_{l,r}|=v_{\max}$ otherwise.

In this work, progressive actuator degradation is evaluated under swarm aggregation movement pattern.

#### 3.4.2 Adaptive sampling pattern

The adaptive sampling movement pattern is a custom movement behaviour introduced in this work, representative of the REMORA use-case for water variable monitoring. Built on top of the random-walk movement pattern where robots alternate between walking and rotating states, the adaptive sampling includes robots autonomously switching between three sampling modes (see Figure 1B): survey, investigation, and high-resolution sampling, with speeds drawn from $v\in \left[v_{\min},v_{\max}\right]$ per mode. Mode transitions are triggered by spatially varying environmental complexities representing distinct zones. These zones are indicative of varying regions of interest for aquatic resource monitoring.

The environment complexity is assumed to be a continuous and evenly distributed spatial field of all three zones. Figure 1B illustrates the varied spatial complexity of water, where red zones characterize high-complexity, yellow for moderate complexity, and lastly blue for low complexity, triggering high-resolution sampling, investigation mode, and survey modes respectively. Sampling modes are governed by fixed complexity thresholds: $c<c_{\text{low}}$ , triggers survey mode, $c<c_{\text{med}}$ , triggers investigation mode, and $c<c_{\text{high}}$ , triggers high-resolution mode. Finally, upon transitioning out of a high complexity zone, a focused sampling mode is triggered with a probability of $p_{\text{foc}}$ , during which the agent remains stationary for a duration of $\Delta t_{dp}$ s for stationary measurement acquisition.

Unlike the aggregation movement pattern, the expected thruster velocities are not fully determined by the instantaneous observation $x_{j}\left(t\right)$ alone. Instead, the observed velocities exhibit a stochastic distribution drawn from $v\in \left[v_{\min},v_{\max}\right]$ , where the underlying sampling mode and observed velocities depend on each robot $r_{j}$ state variables and surrounding environmental conditions (Equation 16), which are unobservable using exteroceptive sensors alone:

$$E\left[v_{j}\left(t\right)|c_{j}\left(t\right)\right]=\left\{\begin{array}{cc} U\left(v_{\text{low, surv}},v_{\text{high, surv}}\right)\text{,} & \text{if }c_{j}\left(t\right)<c_{\text{low}}\;\left(survey\right)\\ U\left(v_{\text{low, inves.}},v_{\text{high, inves.}}\right)\text{,} & \text{if }c_{\text{low}}\leq c_{j}\left(t\right)\leq c_{\text{med}}\;\left(investigation\right)\\ U\left(v_{\text{low, high}-\text{res.}},v_{\text{high, high}-\text{res.}}\right)\text{,} & \text{if }c_{\text{med}}<c_{j}\left(t\right)\;\left(high-resolution\right)\\ 0\text{,} & \text{with probability }p_{\text{foc}}\text{ for }\Delta t_{dp}\;\left(focused.\right) \end{array}\right.$$ (16)

This introduces high temporal ambiguity from the perspective of the observer $r_{i}$ , as it lacks the latent internal variables and environmental readings $c_{j}\left(t\right)$ that precisely dictate the commanded speeds of $r_{j}$ , resulting in velocity profiles and zero-velocity events to appear stochastic. Additionally, the stochastic nature of the underlying random-walk pattern, combined with high spatial complexity, results in a near-infinite combination of state durations and thruster velocities, further increasing the ambiguity of instantaneous features $x_{j}\left(t\right)$ in the absence of temporal context. Consequently, temporal aggregation and descriptive statistics over a time interval are insufficient for identifying complex faults, such as intermittent dropout described in detail in Section 3.3, where specific temporal ordering of events is critical for capturing the latent patterns that characterize such faults.

**FIGURE 3:**
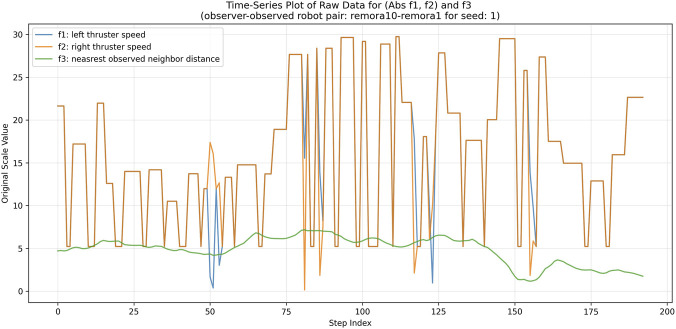
Time-Series plot of features $\left(f_{j}^{1},f_{j}^{2},f_{j}^{3}\right)$ under Adaptive Sampling pattern. Thruster speeds are determined by the complexity of the zone sampled by $r_{j}$ , a feature not observable exteroceptively by the observing robot $r_{i}$ . This makes thruster speeds appear stochastic, further increasing the complexity of temporal fault Modelling.

The complexity of the pattern is demonstrated by plotting the time-series distribution of the its features as shown in Figure 3, where thruster velocities range between $|v_{l,r}|=\left[0,v_{\max}\right]$ cm / s, determined by internal state variables of $r_{j}$ not observable via the exteroceptive sensors.

In this work, intermittent fault is evaluated under adaptive sampling movement pattern.

## 4 Experimental design

### 4.1 Simulation setup and deployment architecture

**FIGURE 4:**
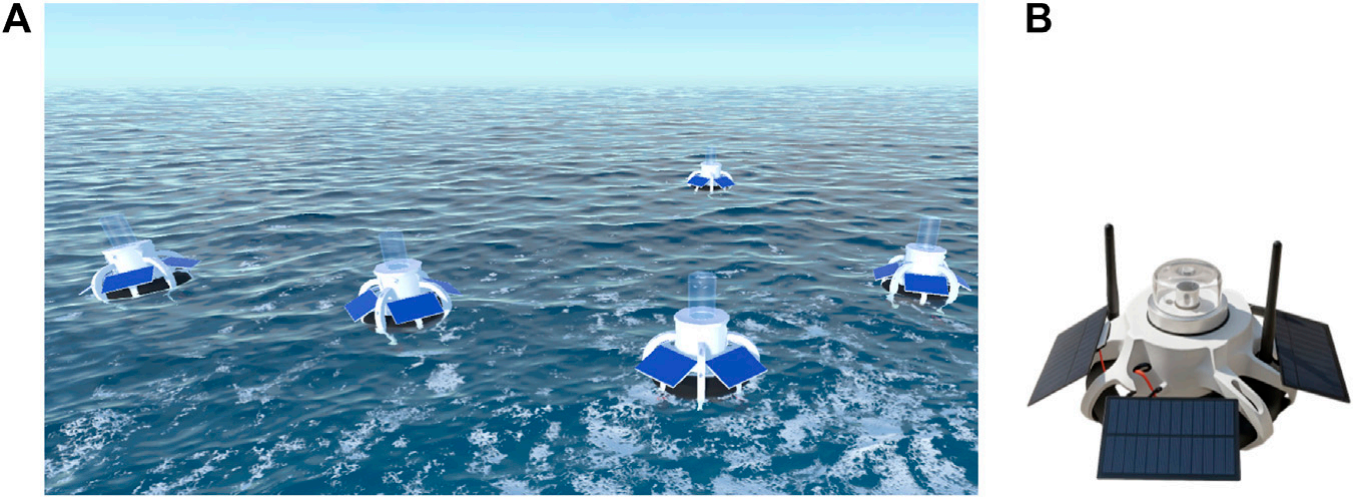
REMORA acquatic simulator for water variable monitoring. **(A)** REMORA bots floating in virtual ocean with simulated wave spectrums, wake generation, and wind-drift. **(B)** REMORA differential-drive USV 3D render.

Experiments were conducted in a custom Unity-based aquatic simulator (see Figure 4A), developed as part of the REMORA project^1^ for aquatic resource monitoring. The REMORA bot shown (see Figure 4B) is a differential-drive unmanned-surface vehicle (USV) whose sensors and actuators interface with the ROS2 middleware via the ROS-TCP-Connector bridge. This enables bidirectional communication between the simulator and ROS2’s computational graph. Swarm behaviours are implemented via the ros2swarm package (Kaiser et al., 2022), allowing for modular, out-of-the-box deployment of distributed swarm movement patterns.

**FIGURE 5:**
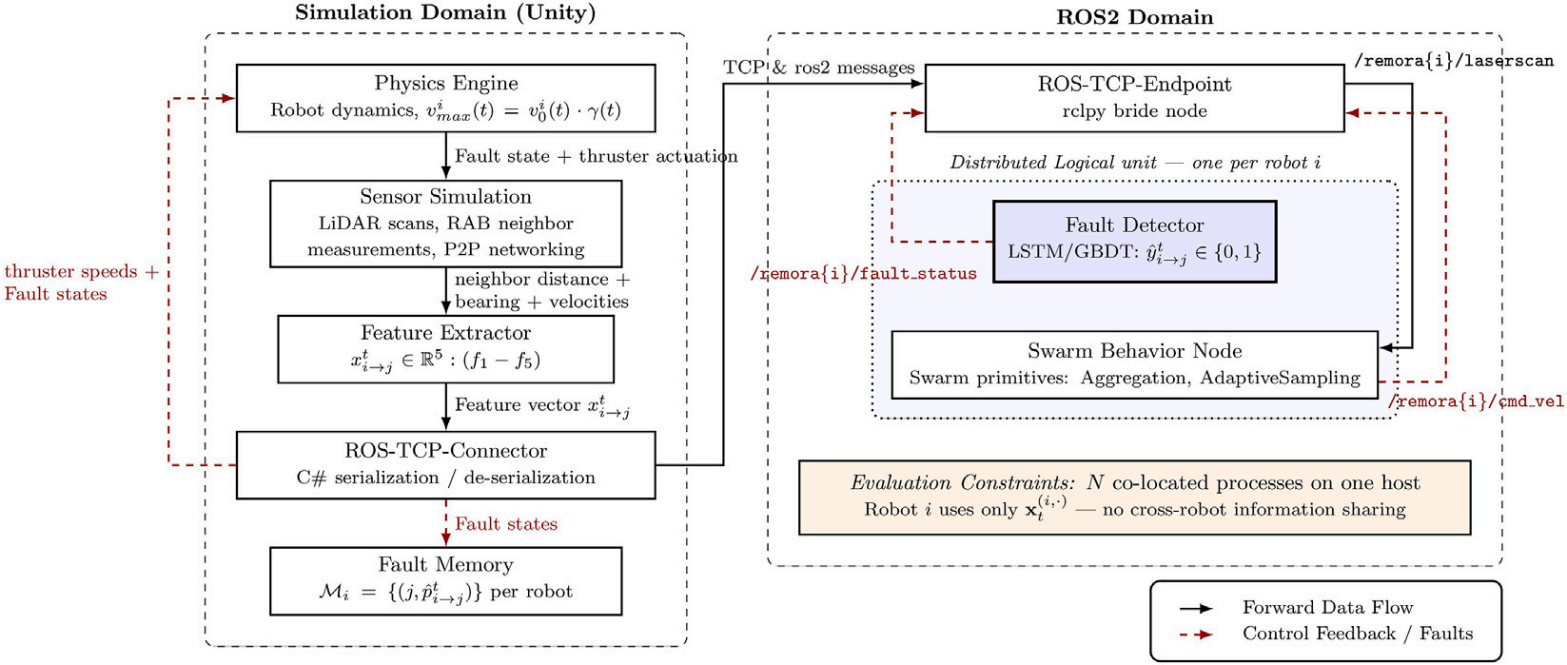
Simulation architecture. overview of the simulation framework, where Unity provides physics simulation while ROS-TCP-Connector bridges the simulator with ROS2 for robotic control and communication.

All Unity and ROS2 processes execute on a single workstation using a single TCP bridge. While the fault detection logic in simulation validation is executed centrally for computational simplicity, the intended architecture will be fully distributed upon real-world deployment: each robot $r_{i}$ subscribes its neighbours $N_{i}\left(t\right)$ and runs a local fault detection node. Per-pair (observer → observed robot pair) metrics are reported in Table 1 to assess the compute feasibility of real-time distributed deployment). Unity serves as the primary engine, driving physics rendering, fault injection, sensor simulation, and feature extraction. The behavioural features $x_{j}\left(t\right)$ are transmitted to the ROS2 computational graph where real-time fault detection is performed via batched inference workers that processes all observed neighbouring robots $r_{j}\in N_{i}\left(t\right)$ in a single forward pass per-robot, per-timestep to reduce latency (batched inference versus single robot inference metrics are also reported in Table 1). Adjacently in the distributed logic unit, the swarm behaviour node processes incoming LiDAR messages to return appropriate commands to Unity for differential-drive actuation of the REMORA bots. The complete system architecture is detailed in Figure 5.

**TABLE 1:** The per observer-observed robot pair deployment metrics for all four models on CPU. Static RAM reports model loading and complete observation history. Total process RAM includes Python runtime, ROS2, and all library dependencies, and peak dynamic RAM per inference call. Latency is reported for both single-pair (batch = 1) and full-neighbourhood (batch = 9) inference.

Model	Task	Disk size (KB)	Static RAM (MB)	Total RAM (MB)	Latency batch = 1 (ms)	Latency batch = 9 (ms)
GBDT	I	19,453	101.7	204.6	3.0±0.8	—
	II	12,986	80.2	181.2	2.9±0.7	—
LSTM	I	51	18.9	785.6	237±24	247±30
	II	273	19.4	786.0	226±29	233±30

The fault detector requires a complete observational buffer of length L to issue a fault prediction ${\hat{y}}_{j}\left(t\right)$ for each neighbour $r_{j}$ . Consequently, each inference worker in the fault detector undergoes a cold-start period of L timesteps at the beginning of new observer-observed robot pair $\left(r_{i}\to r_{j}\right)$ observation sessions, during which no fault prediction is produced. Both LSTM and GBDT architectures operate under identical conditions, isolating fault detection accuracy from any confounding influence from partial or variable-length observation histories.

The feature extractor relies on the communication of thruster velocities $f_{j}^{1}\left(t\right),f_{j}^{2}\left(t\right)$ broadcasted from $r_{j}$ at 10 Hz, consisting of two float32 values (8 bytes). To compute the range and bearing to each neighbour, a 2D pose message from $r_{j}$ is received, consisting of three float64 values (24 bytes), although in simulation the neighbour poses are accessed directly via Unity trigger colliders. These two messages alongside $r_{j}$ ’s ID (uint8) suffice for the computation of all features. More details are described in Carminati et al. (2024); the full implementation is available at: https://github.com/FaisalMazloum/sequential-vs-static-fault-detection-swarm.git (source) and https://doi.org/10.5281/zenodo.20082838 (archived).

The simulation environment consists of a 20×20 ${\text{m}}^{2}$ aquatic arena that is open/obstacle-free, where each of the N=10 homogeneous robotic agents is equipped with a 10 m range-and-bearing (RAB) sensor, within which neighbouring robots are observable, as well as a 10 m 2D-LiDAR for coordinating swarm movement and obstacle avoidance. Each agent within the fleet operates autonomously and decentrally.

### 4.2 Dataset construction

Dataset construction involves a seed-based experimental split, where each seed corresponds to unique simulation runs with distinct MRS spawn locations, thereby creating unique robot behavioural trajectories. A seed split ensures that continuity between each observer-observed robot pair is maintained and prevents any leakage of trajectories into the validation/train sets. Both the LSTM and GBDT architectures use the same seed partitions for training, validation, and testing to ensure a controlled and fair comparison.

#### 4.2.1 Task I: progressive actuator degradation

A total of 200 simulation seeds are used: 100 seeds are reserved for a held-out test set (never used during training or validation), the remaining 100 seeds are split into training and held-out validation sets with an 80/20 ratio. The training underwent K-fold cross-validation for hyperparameter tuning and optimal epoch selection for final retraining. The held-out validation set will be used to select the optimal classification threshold, and the test set will be used to evaluate the model’s performance on unseen trajectories.

Sequences of fixed-length windows ( L=55 s) are constructed using a sliding window with a stride of δt=1 s, generating overlapping sequences for each observer-observed robot pair. Each sequence is standardised and augmented with additive Gaussian noise $\epsilon ∼N\left(\mu ,\sigma^{2}\right)$ to improve robustness and diversity of training samples. This method effectively up-samples the dataset, providing the temporal density necessary for the LSTM to capture long-term dependencies and generalize across varied behavioural trajectories. A many-to-one labelling scheme is applied to each sequence $X_{j}\left(t\right)\in R^{L\times 5}$ ; assigning a single ground truth label of the final timestamp $y_{j}\left(t\right)$ to the entire window. Since degradation is persistent once initiated, all sequences whose final timestep falls after fault injection are labeled as faulty $y_{j}\left(t\right)=1$ , including sequences that predominately contain healthy observations with a final faulty timestep. This ensures detection at the earliest moment of detectable degradation.

The sequential model (LSTM) inputs a sequence of continuous observations $\left(X_{j}\left(t\right)\in R^{L\times 5}\right)$ as a sample, while non-sequential models (GBDT) inputs single observations as a sample $\left(x_{j}\left(t\right)\in R^{5}\right)$ , effectively unfolding a single sequence into L samples, each treated as independent and identically distributed (i.i.d.). This asymmetry is intentional and reflects the fundamental difference in how the two architectures model temporal data. Although flattening continuous observations into high-dimensional feature vectors $R^{L\times d}$ is indeed possible, doing so would incur the curse of dimensionality as the sequence length L grows, a critical constraint in real-world deployment where degradation manifests over days or weeks.

The resulting class distribution is approximately 95:5 (healthy:faulty) for both model architectures.

#### 4.2.2 Task II: intermittent actuator dropout

A total of 650 simulation seeds are used: 150 seeds are reserved for a held-out test set (never used during training or validation), the remaining 500 seeds are split into training and held-out validation sets with an 80/20 ratio. The training set will undergo K-fold cross-validation for hyperparameter tuning and optimal epoch selection for final retraining. The held-out validation set will be used to select the optimal classification threshold, and the test set will be used to evaluate the model’s performance on unseen trajectories.

Unlike progressive actuator degradation, no sliding window upsampling was performed due to the dataset constrains of this fault type. Faults are strictly characterised according to the presence of all three observed precursor criteria and an active dropout as described in Equation 13. A sliding window would result in the loss of precursor information or a shift in the dropout event, invalidating the intermittent fault conditions. Instead, the dataset is constructed from four distinct sample pools, each representing a different behavioural context. All pools use a fixed sequence length of L=51 s: 50 s of precursor information and 1 s to capture whether or not a dropout event occurs at the final timestep. The 50 s of precursor duration is analytically selected, representing the maximum possible duration of the fault precursor observations to manifest (Equation 17):

$$T_{\text{precursor}}^{\max}=3\cdot \Delta t_{\text{turn}}^{\max}+3\cdot \Delta t_{\text{walk}}^{\max}+\Delta t_{\text{walk}}^{\max}=3\cdot 3+3\cdot 8+8=41\text{ s,}$$ (17)

where $\Delta t_{\text{turn}}^{\max}$ and $\Delta t_{\text{walk}}^{\max}$ denote the maximum possible duration of a single turn and walk event respectively. The precursor condition requires three turn-and-walk events to be satisfied: [L,R,L] or [R,L,R], plus an additional walk event for transitioning between zones, yielding a maximum duration of 41 s, rounded to 50 s to provide additional temporal margin. The four pools are labeled using a many-to-one labelling scheme and are defined as follows:

- Pool 1 – Faulty dropout (positive class): Sequence extracted from faulty robots experiencing a voltage-induced ESC shutdown with 50 s of precursor observations. If a faulty robot experiences multiple dropout episodes, each episode generates a separate positive sample provided it is preceded by 50 s of observations. All pool 1 samples are assigned $y_{j}\left(t\right)=1$ .
- Pool 2 – Precursor w/o active dropout (negative class): Sequences extracted from the same faulty robot as pool 1, shifted one timestep earlier to conceal the dropout event. Let $t_{d}$ denote the active dropout event; if pool 1 extracts a sequence $\left[t_{d}-50,\ldots ,t_{d}\right]$ , then pool 2 extracts a window $\left[t_{d}-51,\ldots ,t_{d}-1\right]$ , containing identical precursor observations but without an active dropout event at the final timestep. This ensures that the precursor sequence alone is insufficient for a positive classification. A dropout event must be actively present at the final timestep combined with the precursor information to satisfy the intermittent fault condition in Equation 13. All pool 2 samples are assigned $y_{j}\left(t\right)=0$ .
- Pool 3 – Healthy focused-sampling mode (hard negative class): Sequences extracted from healthy robots performing focused sampling. The robot remains stationary to complete data acquisition of high interest water conditions. This operationalizes Proposition 1: a classifier that models the precursor event and the active dropout will correctly classify the sample as healthy $y_{j}\left(t\right)=0$ . A classifier relying solely on instantaneous halt $\left(v_{l,r}=0\right)$ or aggregated data over a window to detect a dropout event will incorrectly assign $y_{j}\left(t\right)=1$ to these samples, as it is permutation-invariant. This makes the instantaneous models indiscriminate between pools 3 and 1, as both produce identical instantaneous observations. All pool 3 samples are assigned $y_{j}=0$ .
- Pool 4 – Healthy robots with active movement (easy negative class): Sequences extracted from healthy robots that are actively in motion (walking/turning). These constitute the baseline negative class that is easy to distinguish as it contains no active immobilization at current time. All pool 4 samples are assigned $y_{j}\left(t\right)=0$ .

Combining all the pools creates a class distribution of approximately 80:20 (healthy:faulty), where pool 1 constitutes the entirety of the positive class. The remaining pools (2,3,4) constitute the negative class. The inclusion of hard negatives (pool 2 and 3) is intentional as it characterizes the primary discrimination challenge for both model classes, directly testing the theoretical limitations established in Proposition 1.

Similarly to progressive degradation, the sequential model (LSTM) receives the full sequence $\left(X_{j}\left(t\right)\in R^{L\times 5}\right)$ as input, while the non-sequential model (GBDT) receives the unfolded sequences as independent i.i.d. samples $\left(x_{j}\left(t\right)\in R^{5}\right)$ .

### 4.3 Model training and evaluation

Four models are trained and evaluated corresponding to combinations of each architecture (LSTM and GBDT) and each task (progressive degradation and intermittent fault). Each model uses the same seed partitions for training, validation, and testing (discussed in Section 4.2), same hyperparameter search space budget (b=300) , and a fixed random state across all pipeline components, thus ensuring a controlled and fair comparison. Input samples for all models are standardised to align the scales of all features, preventing variables with larger magnitudes from dominating weight updates and speeding up convergence.

Model performance is primarily evaluated on the held-out test set using the $F_{1}$ score. The choice of $F_{1}$ reflects a symmetric cost assumption between false positives and false negatives. This balanced approach is appropriate in the absence of mission-specific cost parameters. In real-mission deployment, these costs are asymmetric: a false positive interrupts mission continuity by triggering needless fault recovery, consequently increasing mission completion time and reducing agent useful life; while missed faults risks mechanical failure and swarm coordination degradation (Winfield and Nembrini, 2006; Bjerknes and Winfield, 2013). We also report the $F_{2}$ score and ROC-AUC alongside $F_{1}$ in Tables 2, 3, where $F_{2}$ penalizes missed faults twice as heavily as false alarms, providing an indicative upper bound on performance under a recall-prioritizingsafety-prioritising cost structure; while ROC-AUC gives a measurement of each classifier’s discriminative ability rather than their performance for an optimally selected threshold. A dedicated analysis of threshold sensitivity and the implications on safety-oriented and mission-preservation-oriented operating points is provided in Section 5.3.

**TABLE 2:** Test set performance for Task I (progressive actuator degradation).

Model	Class	Precision	Recall	$F_{1}$	$F_{2}$	ROC-AUC
LSTM	Faulty	**0.98**	**0.80**	**0.88**	**0.83**	**0.96**
	Healthy	**0.98**	**1.00**	**0.99**	**0.99**	
GBDT	Faulty	0.97	0.67	0.79	0.71	0.95
	Healthy	**0.98**	**1.00**	**0.99**	**0.99**	

**TABLE 3:** Test set performance for Task II (intermittent fault) on the full test set population and a zero-speed subpopulation.

*Full test set*							*Zero-speed subpopulation*	
Model	Class	Precision	Recall	$F_{1}$	$F_{2}$	ROC-AUC	$F_{1}$	ROC-AUC
LSTM	Faulty	**0.93**	**0.98**	**0.95**	**0.97**	**0.99**	**0.95**	**0.99**
	Healthy	**1.00**	**0.99**	**0.99**	**0.99**		**0.96**	
GBDT	Faulty	0.43	0.90	0.58	0.74	**0.99**	0.59	0.56
	Healthy	**1.00**	**0.99**	**0.99**	**0.99**		0.17	

#### 4.3.1 Long short term memory (LSTM)

**FIGURE 6:**
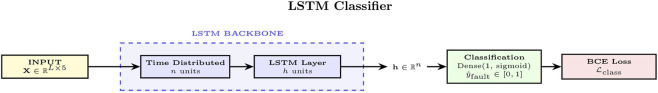
General LSTM architecture. Consists of a time-distributed input to an LSTM layer kernel and recurrent dropout and regularizer, followed by a sigmoid output neuron producing fault probability ${\hat{p}}_{j}\left(t\right)$ . The binary decision ${\hat{y}}_{j}\left(t\right)$ is obtained by thresholding at $\tau^{*}$ .

The LSTM takes a temporal sequence $X_{j}\left(t\right)\in R^{L\times 5}$ as input, where L=55 timesteps for Task I (progressive actuator degradation) and L=51 timesteps for Task II (intermittent fault). These values were chosen to optimize performance and analytically described in Equation 18 to capture sufficient precursor observations. Hyperparameter tuning, paired with 5 fold cross validation was performed to select the best architecture and set of hyperparameters for each task using a Bayesian Optimization Tuner, with the objective of minimizing validation binary cross-entropy (BCE) loss. The optimum set of hyperparameters and architecture by task is presented in Table 4 and Figure 6 respectively.

**TABLE 4:** LSTM hyperparameters for Tasks I and II. Optimum Architecture and hyperparameters selected using BayesianOptimization and 5 fold cross-validation, optimizing for BCE loss.

Hyperparameter	Task I	Task II
Learning rate η	$1\times 10^{-3}$	$3\times 10^{-3}$
Batch size B	2048	2048
L2 Regularization $\lambda_{1}$	$1\times 10^{-5}$	$1\times 10^{-5}$
Recurrent L2 Regularization $\lambda_{2}$	$1\times 10^{-2}$	$1\times 10^{-5}$
Kernel dropout rate κ	0.6	0.6
Hidden units h	4	52
Input Dense units n	62	38
Optimum epoch $e^{*}$	106	108
Optimum classification threshold $\tau^{*}$	0.70	0.59

Class imbalance is addressed by applying balanced class weights $w_{\mathrm{class}}=N/\left(K\cdot n_{\mathrm{class}}\right)$ , where N denotes the total number of training samples in the dataset, K for the total number of distinct classes, and $n_{\mathrm{class}}$ for the total number of class training samples. The Adam optimizer was used with gradient clipping at threshold $\tau_{g}=1.0$ to prevent exploding gradients. The model was tasked with minimizing binary cross-entropy (Equation 18):

$$L_{\mathrm{BCE}}=-\frac{1}{B}\overset{B}{\underset{i=1}{\sum }}w_{class_{i}}\left[y_{i}\log\left(\hat{p_{i}}\right)+\left(1-y_{i}\right)\log\left(1-{\hat{p}}_{j}\right.\right]\text{,}$$ (18)

where $w_{class_{i}}$ denotes the class weight and ${\hat{p}}_{j}$ the predicted fault probability for sample i .

The optimal training epoch $e^{*}$ is determined via 5 fold cross-validation on the training set, grouped by training seed to prevent data leakage. Each training fold consisted of two callback methods: early stopping after 40 epochs without improvements and learning-rate reduction on plateau, triggered after 12 epochs without improvement to stabilize convergence. The mean BCE validation loss curve is computed, where $e^{*}$ is defined as (Equation 19):

$$e^{*}=\arg\min_{e}\;\frac{1}{K}\overset{K}{\underset{k=1}{\sum }}L_{\text{val\_BCE}}^{k}e\text{,}$$ (19)

where $L_{\text{val\_BCE}}^{k}e$ denotes the validation BCE loss at epoch e for fold k . The final model is retrained on the full training dataset (train plus validation set) for exactly $e^{*}$ epochs with no early stopping callback. Tasks I and II yield $e^{*}=106$ and $e^{*}=108$ respectively.

The optimum classification threshold $\tau^{*}$ is selected by maximizing the score $F_{1}$ on the held-out validation set from the precision-recall curve, yielding $\tau^{*}$ = 0.70 for Task I and $\tau^{*}$ = 0.59 for Task II. The full set of optimal parameters are presented in Table 4.

#### 4.3.2 Gradient boosted decision tree (GBDT)

GBDT takes single timestep observations $x_{j}\left(t\right)\in R^{5}$ as input, treating each as an i.i.d. sample. Hyperparameter tuning paired with 5 fold cross validation was performed to select the best architecture and set of hyperparameters for each task using a random search with a budget of 300 configuration combinations, with the objective of minimizing validation binary cross-entropy (BCE) loss. The optimum set of hyperparameters by task is presented in Table 5.

**TABLE 5:** GBDT hyperparameters for Tasks I and II. Optimum Architecture and hyperparameters selected using a random search and 5 fold cross-validation, optimizing for BCE loss.

Hyperparameter	Task I	Task II
Learning rate	0.1	0.05
n_estimators	500	400
Subsample	0.9	0.9
min_samples_split	30	20
min_samples_leaf	5	10
max_features	sqrt	sqrt
max_depth	9	9
Optimum classification threshold $\tau^{*}$	0.85	0.28

Similar to LSTM’s training procedure, class imbalance is addressed using class weights and the optimal threshold $\tau^{*}$ is selected to optimize the $F_{1}$ score on the held-out validation set from the precision-recall curve, yielding $\tau^{*}=0.85$ for Task I and $\tau^{*}=0.28$ for Task II.

## 5 Results and discussion

Four models are evaluated in this work for their reliability in exogenous detection of complex, time-dependent faults: LSTM versus GBDT under progressive actuator degradation (Task I), and LSTM versus GBDT under intermittent fault caused by ESC dropout (Task II). Additionally, threshold sensitivity to operating point selection is also analysed, and the computational feasibility of all models for real-time distributed deployment is evaluated and reported in this section.

### 5.1 Task I: fault detection reliability under progressive actuator degradation

Table 2 reports the test set performance of LSTM and GBDT on progressive actuator degradation fault type, the simpler temporal problem of the two tasks. The LSTM achieves an $F_{1}$ score of 0.88, outperforming GBDT by 11.4%. This performance gap is mainly driven by recall, where LSTM correctly identifies 80% of all faulty robots compared to 67% by GBDT. Both models achieved high ROC-AUC scores of approximately 0.95, indicating high discriminative ability across thresholds, and high precision of 0.95, indicating highly reliable faulty classifications.

**FIGURE 7:**
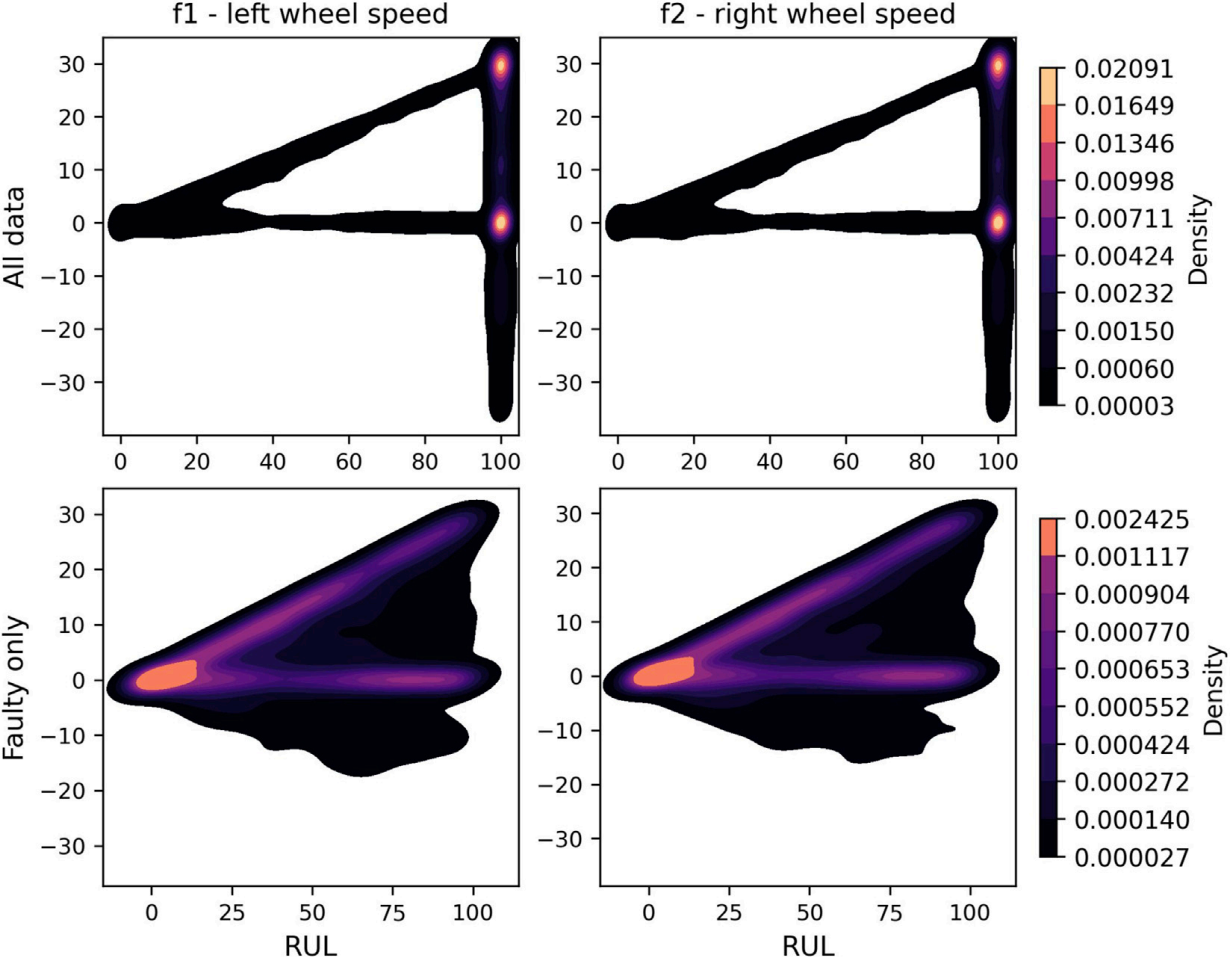
Kernel Density Estimation (KDE) plots of thruster speeds for all robots (Row 1 – All data) and faulty only robots (Row 2 – Faulty only) robots. Healthy robots are denoted with remaining useful life (RUL) = 100, at which thruster velocities concentrate at $v=v_{\max}=30$ cm / s in states $S_{j}\left(t\right)=\left\{1,2,3\right\}$ (operating at max thrust capacity), and v=0 in state $S_{j}\left(t\right)=4$ (cohesion state). Faulty robots are denoted with remaining useful life (RUL) ≠ 100, at which maximum thruster velocities never reach $v=v_{\max}=30$ cm / s due to the degradation in maximum possible capacity.

Both architectures yield strong performance. The similarity in performance could be explained by the deterministic nature of the aggregation pattern paired with progressive linear degradation. Figure 7 shows the concentration of thruster speeds across all remaining useful life, where RUL=100 represents healthy robot behaviour that has not yet experienced any form of degradation. At these points, healthy robots have two heavy concentrations, particularly constant non-zero speeds $v=v_{\max}$ (assuming no obstacles to avoid) at states $S_{j}\left(t\right)\in \left\{1,2,3\right\}$ , representing movement states, and v=0 at $S_{j}\left(t\right)\in \left\{4\right\}$ representing cohesion state. Any deviation below $v_{\max}$ as caused by degradation is therefore indicative of fault, as demonstrated in Figure 7 where the maximum capacity of the thruster speeds decrease as RUL decreases, creating a distinct signal for the models to leverage.

The primary differentiator in performance stems from the zero-speed population, a value overlapping in both healthy and faulty classes as shown in Figure 7. Zero-speed events are triggered if $f_{j}^{3}\leq d_{\text{coh}}$ (as discussed in Section 3.4.1), and remain stationary for a predefined duration. In this state, GBDT lacks the motion trajectory of $r_{j}$ to determine if it sustained a speed of $v<v_{\max}$ prior to entering cohesion, defaulting to classifying all cohesion states as healthy. This region of ambiguity explains the gap in recall between the two models: LSTM achieves a recall of 0.80, whereas GBDT achieves 0.67, indicating that a large portion of faults go unnoticed. While LSTM detects majority of fault events, its performance is constrained by a specific temporal sequence: a robot may maintain a cohesion state for a duration exceeding the model’s fixed-length window. In such instances, the fault signal characterised by $v<v_{\max}$ is diluted by cohesion signals (v=0) , which dominate the input window. However, this constraint is not a lack of model capacity, but rather a suboptimal architectural configuration. Performance can be improved by extending the window length to encompass a longer historical context.

These analyses reveal that for most of the robot’s remaining useful life (RUL>0) , identifying aggregation faults remains a relatively simple temporal problem, i.e., instantaneous features are sufficient for reliably identifying faulty robots in active-motion. For behaviourally ambiguous regions (v=0) , where both healthy and faulty robots have identical behavioural information, the GBDT baseline model lacks the temporal context to distinguish healthy from faulty robots.

### 5.2 Task II: fault detection reliability under intermittent faults

Table 3 reports the test set performance of LSTM and GBDT on intermittent fault type, the more complex temporal problem of the two tasks. The evaluation metrics consist of two populations: a population containing all four pools (described in Section 4.2) representing the full dataset, and a subpopulation containing only pools 1 and 3: the faulty dropout and healthy stationary robots. This subpopulation directly tests the indistinguishability argument of Proposition 1, where the instantaneous observations $x_{j}\left(t\right)$ of both pools are identical, and only temporal context $X_{j}\left(t\right)$ carries discriminative information.

On the complete test set (all four pools), LSTM achieves an $F_{1}$ score of 0.95, significantly outperforming GBDT with a score of 0.58. The low performance of GBDT is directly a function of its precision-recall asymmetry: precision of 43% and recall of 90%, demonstrating over-classification of faults. Additionally, both models achieved near-perfect ROC-AUC scores (0.99). However, this does not conclusively reflect each model’s true discriminative ability, given the heavy pool imbalance of the complete test set.

As per the zero-speed subpopulation comprising of pools 1 and 3, representing the positive and hard negative classes respectively, LSTM maintains its $F_{1}$ and ROC-AUC performance ( $F_{1}$ = 0.95 and ROC-AUC = 0.99), while GBDT’s ROC-AUC score plummets to 0.56, equivalent to random guessing. This demonstrates that the LSTM has discriminative ability regardless of pool imbalance, whereas the baseline GBDT’s residuals are primarily driven by the non-zero-speed subpopulation.

The GBDT baseline suffers a severe precision-recall asymmetry on intermittent faults (precision of 0.43 and recall of 0.90), reflecting the model’s inability to distinguish between specific pools. At pools 2 (precursor without dropout) and 4 (healthy robot with non-zero speeds), samples are accurately assigned $\hat{y_{j}}\left(t\right)=0$ , as non-zero thruster speeds are trivially classified as healthy. However, faced with zero-speed subpopulations: pools 1 (faulty robot with dropout) and 2 (hard negative class, healthy robot with zero-speed), non-sequential models cannot leverage the ordered precursor sequence to determine whether a halt is caused by an ESC-induced voltage shutdown or intentional for data acquisition (focused sampling mode), and thereby defaulting to classifying all zero-speed events as faulty. This default towards classifying as faulty is a direct consequence of class-weighted training. Since the positive class (pool 1, faulty robot) is the minority class, the balanced class weights assign higher loss penalties to missing faults than to false-positives. On the complete dataset, comprising all four pools, this method is appropriate as it prevents the model from ignoring the minority class. However, in the zero-speed subpopulation (pool 1 and pool 3), where pool distribution is approximately 1:1, the expected false positives become more pronounced as shown in Table 3.

The near-perfect ROC-AUC of 0.99 achieved by GBDT on the complete dataset is misleading as a standalone performance indicator. ROC-AUC measures the models discriminative ability across all classification thresholds, and is heavily influenced by the presence of easy negatives, specifically the high density of easy negatives (pools 2 and 4).

**FIGURE 8:**
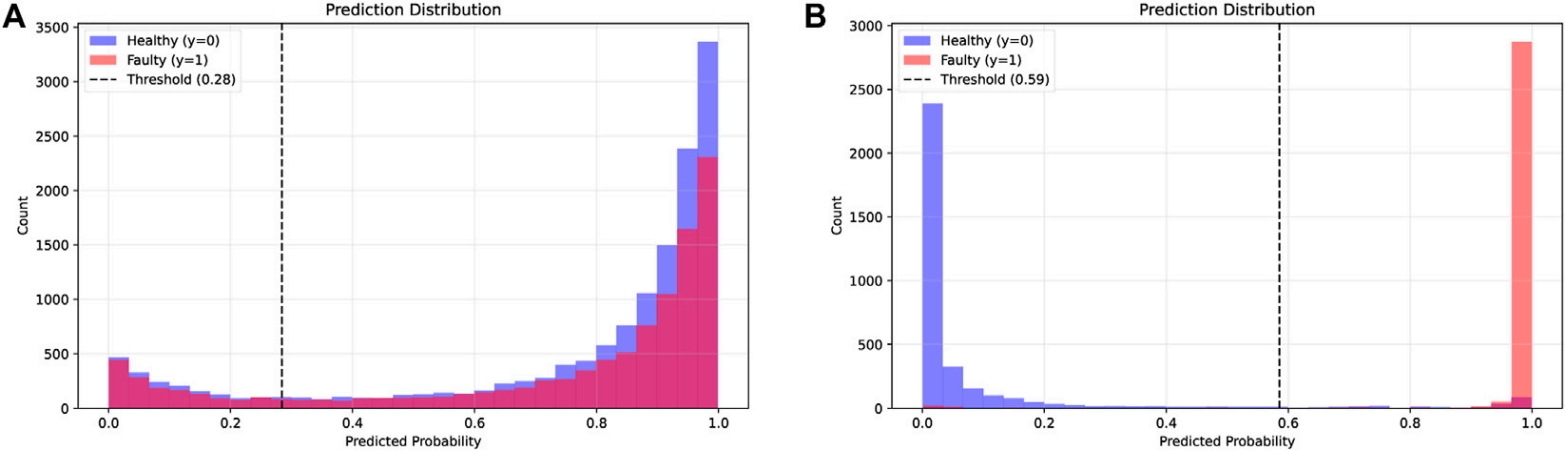
Prediction distribution of GBDT and LSTM under zero-speed subpopulations with intermittent fault type. **(A)** GBDT showing faulty and healthy predictions heavily overlap regardless of selected classification threshold. **(B)** LSTM showing clear discriminative ability between faulty and healthy robots.

The zero-speed subpopulation results confirm these interpretations. By constraining the evaluation pools to 1 and 3 where instantaneous observations are identical, the GBDT baseline model’s ROC-AUC plummets to 0.56, equivalent to random guessing. This confirms that the optimistic scores for healthy classification and discriminative ability is entirely attributed to easy negatives. Regardless of classification threshold, a low ROC-AUC indicates unreliable separation between faulty and healthy stationary robots, as illustrated in Figure 8A, which shows a significant overlap of predicted scores across all thresholds.

On the other hand, LSTM maintains its performance $\left(F_{1}=0.95,\text{ROC}-\text{AUC}=99\%\right)$ , revealing that strict ordered precursor sequence and dropout event (as described in Equation 13) is sufficient and necessary for reliable complex fault detection.

These results empirically validate Proposition 1: non-sequential model’s inability is not a consequence of capacity or tuning limitations, rather an architectural limitation of permutation invariance under a fault type whose discriminative signature is temporally ordered.

Supplementary videos demonstrating the fault detection framework under both fault types are available online (see Supplementary Material).

### 5.3 Threshold sensitivity and operating point analysis

**FIGURE 9:**
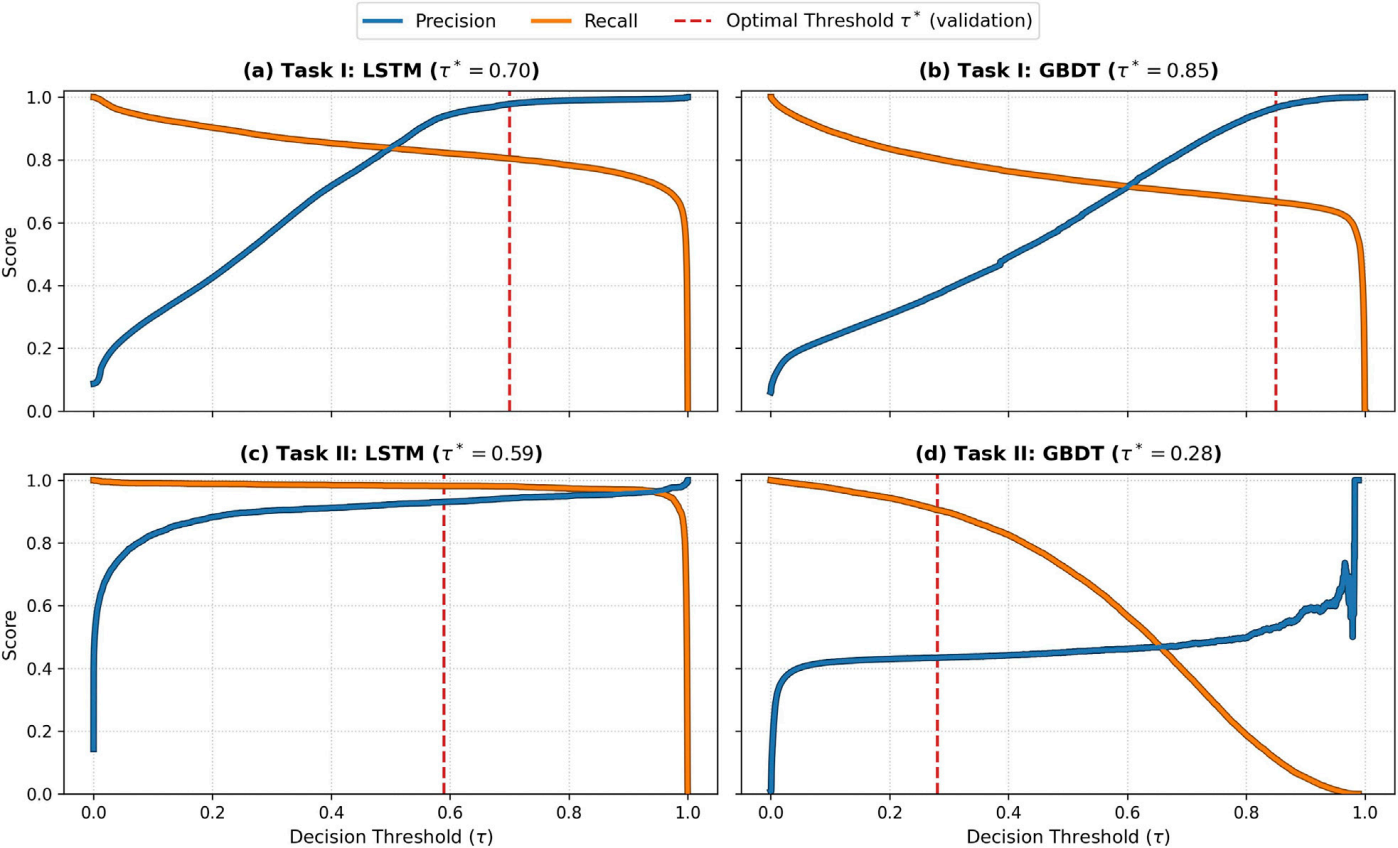
Precision and recall as a function of decision threshold τ for all four models. Panels **(a,b)** Task I (progressive actuator degradation) for LSTM and GBDT respectively. Panels **(c,d)** Task II (intermittent fault) for LSTM and GBDT respectively. The vertical dashed line marks the validation-set-optimal threshold $\tau^{*}$ in each panel. Panel **(c)** shows that LSTM Task II performance is stable across safety-oriented (low τ ) and mission-preservation-oriented (high τ ) operating points. Panel **(d)** shows that GBDT precision is capped at ∼0.40 across the full mid-range, confirming that the performance failure is distributional and not recoverable by threshold adjustment.

This subsection analyses the sensitivity of model conclusions to threshold selection and evaluates performances across safety-oriented and mission-preservation-oriented operating points. Figure 9 plots precision and recall as a function of decision threshold τ for all four models across both tasks.

For Task I (panels a–b), both architectures exhibit similar precision-recall trade-off behaviour across the full threshold range: recall remains approximately stable across the mid range τ∈[0.1,0.95] , while precision rises approximately linearly with τ . Lowering τ towards a safety-oriented operating point marginally increases recall, but at the expense of a substantial increase in false alarms, as shown by the relatively sharp drop in precision. This asymmetry implies that shifting towards a safety-oriented operating point is only justifiable when the operational cost of a missed-fault substantially outweighs that of a false alarm. In less critical scenarios, the validation-selected optimal threshold $\tau^{*}$ represents a more operationally efficient operating point, capturing majority of fault events while limiting unnecessary mission interruptions.

For Task II (panels c–d), the two architectures diverge sharply. Panel (c) shows that LSTM maintains near-perfect precision and recall across the full mid-range τ∈[0.1,0.95] . This indicates strong discriminative ability, such that the choice of operating point has negligible impact on either metric. A mission operator can freely select any threshold within this range, whether safety-oriented or mission-preservation-oriented, without meaningful degradation in performance. On the contrary, Panel (d) reveals operationally problematic behaviour for GBDT: precision plateaus at ∼0.40 across the entire mid-range regardless of τ , indicating that the model produces excessive false alarms that no threshold adjustment can suppress, while recall monotonically degrades as τ increases. Adjusting the threshold towards a mission-preservation-oriented operating point yields no precision improvement, and critically only increases missed faults. Threshold selection therefore offers no viable operating point for GBDT on Task II, consistent with the ROC-AUC of 0.56 reported for the zero-speed subpopulation in Table 3.

### 5.4 Computation feasibility of distributed deployment

To validate the feasibility of the intended distributed deployment architecture, the per observer-observed robot pair $\left(r_{i}\to r_{j}\right)$ compute metrics are evaluated, specifically: inference latency, model memory footprint, and process RAM for all four models on CPU, reflecting conservative onboard compute for preliminary REMORA USV deployment. Benchmarks were conducted using 1000 single-sample inferences with 100 warmup calls.

GBDT models occupy significantly larger disk space (13–19 MB) and static RAM (80–102 MB), primarily due to the storage of 400–500 decision trees with max _ depth = 9. However, LSTM requires significantly higher total process RAM ( ∼ 780 MB) due to the overhead of the implemented deep learning framework. Dynamic RAM per inference call is negligible for both architectures: ∼6 KB and ∼ 80 KB for GBDT and LSTM respectively, confirming per-inference memory overhead does not constrain deployment.

Both architectures report per-pair CPU latency below Δt=1 s: ∼3±0.8 ms for GBDT models versus ∼230±25 ms for LSTM. Moreover, LSTM with full neighbourhood batched inference (batch = 9) yields identical inference latency to single batched inference (batch = 1) in a single forward pass ( ∼235±30 ms). While per-pair cost scales linearly with $\left|N_{i}\left(t\right)\right|$ , batched workers eliminate this scaling in practice, keeping per-inference call latency below the observational timestep increment Δt .

Both architectures satisfy the real-time constraint of Δt=1 s. In terms of memory, GBDT’s total process RAM of ∼ 181–205 MB and LSTM with a ∼ 780 MB footprint are both compatible with higher-end embedded computers such as the NVIDIA Jetson series.

## 6 Conclusions, limitations, and future work

This work investigated whether memoryless, non-sequential models can provide reliable exogenous fault detection for temporally complex fault types in distributed multi-robot systems, and whether recurrent neural networks offer an architectural advantage over non-sequential baselines. Two fault types were evaluated in this work under unique swarm movement patterns: progressive actuator degradation under swarm aggregation and intermittent actuator dropout under a custom Adaptive Sampling pattern. Each fault type and movement pattern pair represents a distinct category of temporal complexity in observed feature signatures.

Results reveal that non-sequential models provide mostly reliable fault detection of progressive actuator degradation, where the deterministic behaviour of agents performing aggregation limits observational ambiguity. However, for intermittent faults, non-sequential models degrade to chance level performance, particularly for zero-speed subpopulations where feature ambiguity is highest. This results in excessive false alarms, leading to needless task interruptions, effectively slowing down mission completion and wasting the remaining useful life of the robot in the process. LSTM provides consistent high performance across both tasks, due to the structural advantage of leveraging temporal ordering that non-sequential models cannot access without explicit feature engineering. To conclude, sequential models are essential when the discriminative signature of a fault is defined by temporally ordered precursors, whereas memoryless models can be sufficient when instantaneous features or their windowed aggregates uniquely characterize the fault state.

The main contributions of this work are threefold. First, we provide a formal impossibility result (Proposition 1) establishing that memoryless classifiers are provably insufficient for fault types whose discriminative signature depends on temporally ordered precursors, a theoretical grounding absent from prior exogenous fault detection studies in multi-robot systems. Second, we present the first empirical evaluation of sequential behavioural modelling for complex, time-dependent fault types in distributed multi-robot systems, demonstrating that LSTM operating on generic, primitive features achieves reliable exogenous detection without domain-specific priors. Third, we provide compute benchmarks confirming that both architectures satisfy real-time constraints and are compatible with embedded onboard hardware, bridging the gap between simulation evaluation and practical distributed deployment.

The practical significance of these contributions is grounded in the prevalence of time-dependent fault types in real marine robotic deployments: progressive actuator degradation caused by biofouling or bearing wear develops over days to weeks, while intermittent dropout arises from thermally or mechanically induced ESC instability. In both cases, detection failures lead to needless task interruptions, increased repair costs, and reduced fleet uptime. The results demonstrate that deploying a memoryless fault detector in such settings is suboptimal: for intermittent faults, it degrades to chance-level performance, rendering the detector operationally useless. Sequential models are therefore not a marginal improvement but a necessary architectural requirement for fault-tolerant multi-robot systems operating in realistic field conditions.

This work has several limitations. Experiments are conducted in simulation only, using idealised fault models with a fixed swarm size of N=10 and specific movement pattern and fault type pairings. The fault detector currently assumes that all observed robots $r_{j}\in N_{i}\left(t\right)$ remain continuously within RAB sensor range for the full observation window of length L ; if a robot exits sensor range mid-sequence, the observation buffer is invalidated and the cold-start period of L timesteps must restart upon re-entry. Additionally, the threshold selection optimises $F_{1}$ under a symmetric cost assumption between false positives and missed detections, which may not reflect real mission priorities.

This work has several limitations:

- Simulation-only evaluation. Experiments are conducted in simulation only, using idealised fault models with a fixed swarm size of N=10 and specific movement pattern and fault type pairings. Generalization to real hardware, variable swarm sizes, environmental disturbances and uncertainty remains to be validated.
- Observation history completeness and consistency. The fault detector assumes that all observed robots $r_{j}\in N_{i}\left(t\right)$ remain continuously within RAB sensor range for the full observation window of length L ; if a robot exits sensor range mid-sequence, the observation buffer is invalidated and the cold-start period of L timesteps must restart upon re-entry. However, this addresses only the case of complete observation loss through range exit. A more pervasive failure mode is data loss and message intermittency within an active observation window, which will occur even when robots remain spatially proximate. In marine environments, wireless communication is subject to multipath fading, wave-induced attenuation, and electromagnetic interference from onboard electronics, all of which can produce dropped or delayed messages without any change in inter-robot distance. A single missing timestep within an ongoing sequence corrupts the input tensor $X_{j}\left(t\right)\in R^{L\times 5}$ , requiring either imputation of the missing observation or invalidation of the entire window, neither of which is evaluated in this work.
- Threshold selection and symmetric cost assumption. The decision threshold is selected by optimising $F_{1}$ on the validation set, reflecting a symmetric cost assumption between false positives and missed detections. As analysed in Section 5.3, this may not reflect real mission priorities, where the operational cost of a missed fault and a false alarm are rarely equal.

Future work will involve four directions. First, non-sequential models will be evaluated with explicit temporal aggregates and descriptive statistical features (lagged values, sliding-window statistics) to isolate whether the LSTM advantage in multi-robot fault detection is architectural or feature-driven, using a more complex mission design that requires inferring the ongoing task from exteroceptive features. Second, the framework will be extended toward a complete fault detection, diagnosis, and recovery (FDDR) pipeline, wherein the fault detection threshold is adjusted autonomously based on estimated remaining useful life (RUL) and distance to the nearest maintenance station, enabling predictive maintenance and graceful degradation. Third, asymmetric cost weighting of the form L=α⋅FPR+β⋅FNR with domain-calibrated parameters derived from real mission constraints will be incorporated, enabling threshold selection that explicitly accounts for the relative cost of false alarms versus missed faults. Fourth, partial or interrupted observation histories, arising when observed robots temporarily exit sensor range, will be addressed via buffer imputation or variable-length sequence models. incomplete, delayed, or inconsistent observational histories, arising from observed robots temporarily exiting sensor range, message dropout, or communication intermittency within an active observation window, will be addressed via buffer imputation or variable-length sequence models.

## Data Availability

The datasets presented in this study can be found in online repositories. The names of the repository/repositories and accession number(s) can be found in the article/Supplementary Material. Supplementary videos demonstrating the fault detection framework under both fault types are available in the Supplementary Material.

## Footnotes

1 REMORA: Swarm Robotics for the Observation and Enhancement of Aquatic Resources (COMPETE2030-FEDER-01429400). https://remora.ingeniarius.pt/.
